# Implementation of an institutional protocol for rational use of blood products and its impact on postoperative of coronary artery bypass graft surgery

**DOI:** 10.1590/S1679-45082013000300009

**Published:** 2013

**Authors:** Pedro Gabriel Melo de Barros e Silva, Dimas Tadahiro Ikeoka, Viviane Aparecida Fernandes, Nilza Sandra Lasta, Debora Prudencio e Silva, Mariana Yumi Okada, Beatriz Akinaga Izidoro, José Carlos Teixeira Garcia, Antonio Claudio do Amaral Baruzzi, Valter Furlan

**Affiliations:** 1 Amil Clinical Research/Hospital TotalCor, São Paulo, SP, Brazil

**Keywords:** Thoracic surgery, Myocardial revascularization, Blood transfusion, 6-aminocaproic acid, Hemorrhage

## Abstract

**Objective:**

Cardiac surgeries are sometimes followed by significant blood loss, and blood transfusions may be necessary. However, indiscriminant use of blood components can result in detrimental effects for the patient. We evaluated the short-term effects of implementation of a protocol for the rational use of blood products in the perioperative period of cardiac surgery.

**Methods:**

Between April and June 2011, an institutional protocol was implemented in a private hospital specializing in cardiology to encourage rational use of blood products, with the consent and collaboration of seven cardiac surgery teams. We collected clinical and demographic data on the patients. The use of blood products and clinical outcomes were analyzed during hospital stay before and after protocol implementation. The protocol consisted of an institutional campaign with an educational intervention to surgical and anesthesiology teams; the goal was to tailor blood transfusion practice according to clinical goals (anemia with hemodynamic changes and significant ventricular dysfunction) and to make routine the prescription of Ɛ-aminocaproic acid intraoperatively, which is recommended by international guidelines based on scientific evidence.

**Results:**

After three months of protocol implementation, the use of Ɛ-aminocaproic acid increased from 31% to 100%. A total of 67% of surgeries before protocol implementation required any blood transfusion, compared with 40% that required any blood transfusion after protocol implementation in subsequent months of the same year (p<0.001). There was no significant difference in clinical outcomes assessed before and after implementation of the protocol.

**Conclusion:**

The rational use of blood products associated with infusion of Ɛ-aminocaproic acid has the potential to reduce the number of blood transfusions in perioperative of cardiac surgeries, but it can affect the risk of complications.

## RESUMO

**Objetivo:**

Cirurgias cardíacas são, por vezes, acompanhadas de perdas sanguíneas significativas, e transfusões de sangue podem ser necessárias. No entanto, o uso indiscriminado de hemoderivados pode resultar em efeitos danosos para o paciente. Neste estudo, avaliamos os efeitos imediatos da implantação de um protocolo para o uso racional de hemoderivados no perioperatório de cirurgias de revascularização miocárdica.

**Métodos:**

Entre os meses de abril e junho de 2011, foi implementado um protocolo institucional em um hospital privado especializado em cardiologia com a anuência e a colaboração de sete equipes de cirurgia cardíaca, visando ao uso racional de hemoderivados. Foram verificados dados clínicos e demográficos dos pacientes, e avaliados o uso de hemoderivados e os desfechos clínicos no período intra-hospitalar, antes e após a implantação do protocolo. O protocolo consistiu em uma campanha institucional junto às equipes cirúrgicas, de anestesiologia e intensivistas, para difundir a prática do uso de hemoderivados com base em critérios clínicos objetivos (anemia com repercussões hemodinâmicas e disfunção ventricular significativa), bem como tornar rotineira a prescrição de ácido epsilon-aminocaproico no intraoperatório, que é prática recomendada por diretrizes internacionais baseadas em evidência científica.

**Resultados:**

Após os 3 meses de implantação do protocolo, houve aumento do uso de ácido epsilon-aminocaproico de 31% para 100%. Antes da implantação do protocolo, 67% das cirurgias utilizaram alguma transfusão sanguínea; após a implantação, 40% das cirurgias necessitaram de alguma transfusão sanguínea nos meses subsequentes do mesmo ano (p<0,001). Não houve diferença significativa nos desfechos clínicos avaliados antes e após implantação do protocolo.

**Conclusão:**

O uso racional de hemoderivados, associado à infusão do ácido epsilon-aminocaproico, tem o potencial de reduzir o número de hemotransfusões no perioperatório de cirurgias cardíacas, o que pode ter impacto no risco de complicações.

## INTRODUCTION

Heart surgeries are great procedures associated with high risk of bleeding. Several causes are often described as hemorrhagic complications in postoperative of cardiac surgery, such as preexisting coagulation disorders or caused by surgical trauma and extra-corporal circulation (ECC), hyperthermia, hemodilution, use of intraoperative heparin, technical factors related with the procedure and handling of large vessels^(1–3)^. Acute anemia from significant blood loss may lead to a reduction of oxygen provided to myocardium, therefore, increasing the chances of unfavorable evolution that justify, in several cases, the indication of blood transfusions^(4,5)^. According to large reports, more than 50% of patients submitted to heart surgeries received transfusion of high variability. In addition, these studies stated that blood products were used in more than 90% of cardiac surgeries^(6–9)^. In the United States roughly 20% of all erythrocyte concentration collected are used in cardiac surgeries^(6–9)^.

Although the use of blood products is well tailored, the literature often related it to high risk of infection, renal impairment, hospital mortality and even late mortality^(10–13)^. In addition, normal hemoglobin levels or close to normal are not necessarily associated with better evolution in the postoperative period, which suggest an advantage in avoiding blood transfusions and tolerating lower hemoglobin levels, but this index could not be followed by hypotension or evidences of reduction on tissue perfusion^(14)^. Some studies report that half of transfusion in patients submitted to heart surgery is unnecessary or inappropriate^(15)^. As a result, in such cases, to adopt adequate clinical and laboratory criteria to indicate blood products transfusion in order to reduce the risk of complications seems to be an advantage. An institutional protocol following guidelines based on evidences^(15,16)^ can guide better this condition.

Our hypothesis is that to use educational interventions related to the rational use of blood products, and to turn the use of antifibrinolytics a routine may reduce the number of transfusion in perioperative period of cardiac surgery.

## OBJECTIVE

To evaluate the short-term effects of protocol implementation for the rational use of blood products in perioperative period of major cardiac surgeries in a hospital specialized in the treatment of cardiovascular diseases.

## METHODS

We used a database of cardiac surgeries of the *Hospital TotalCor*, located in the city of São Paulo (SP), Brazil. The hospital is specialized in treating cardiovascular disease and has 97 beds. The comparative analysis included patients who underwent isolated myocardial revascularization surgery because they constitute a more homogeneous group with regarding demographic characteristics, which is different from other types of cardiac surgery.

Patients submitted to myocardial revascularization surgery before the protocol implementation (January to March 2011) was compared with the group of patients included in July to December 2011, i.e., after the protocol implementation that occurred between April and June 2011.

The institutional protocol of the rational use of blood products was set of measures that counted with consent and collaboration of seven cardiac surgical team that conducted surgeries in the hospital. The protocol was composed by an educational campaign with surgical and anesthetic teams as well as with intensive therapy team. To diffuse the practice of blood products use it was based on clinical criteria prior defined along with department of hematology and hemotherapy of the institution that used scientific evidences appointed to create the guidelines. The protocol aimed to avoid indication of transfusions based only in isolated values of hematocrit and hemoglobin and also to turn the prescription of epsilon aminocaproic acid intraoperative (EAAI) as routine in the intraoperative period. Therefore, we defined in institutional protocol the eligibility criteria to perform transfusions of each blood products, including:

1. erythrocyte concentration (A – symptomatic anemia without specific treatment and serum hemoglobin <10g/dL; B – hemoglobin <7g/dL in asymptomatic patient in the perioperative period; C – hemoglobin between 7 and 10g/dL in patients with risk to cardiac ischemia in preoperative period; D – hemoglobin <11g/dL in patients with unstable coronary disease; E – hemoglobin <10g/dL for patients in clinical situations with risk more elevated for bleeding or low intraoperative tissue perfusion, falciform anemia, thalassemia, age over 65 years old, etc.; F – acute anemia caused by bleeding with clinical criteria of low tissue perfusion such as tachycardia, hypotension, late capillary refill, tachypnea, low urinary output, altered mental status);
2. platelet concentration (A – active bleeding with platelets counting <50 mil/mm^3^; B – platelet dysfunction with active bleeding; C – platelet counting lower than 20 thousand associated with chemotherapy, tumor invasion, leukemia or bone marrow aplasia);
3. fresh frozen plasma (A – active bleeding followed by multiple coagulation factor deficiency; B – hepatopathy patients with international standard index (ISI) >1.5 and with signals of active bleeding or in preoperatory period).

Others blood products were not used (total blood) or were used with minimal frequency, which cause an exclusion of them from our analysis. The amount of each blood product to be used in eligible patients was included in institutional protocol and followed the international recommendation^(15,16)^. The surgical team registered to conduct the procedure as well as anesthetists and intensivists of the institution participated in meetings with protocol managers team, participants were instructed about criteria for blood products prescription and administration. They were also encouraged to follow prior recommendations of the protocol established by the blood bank. It was recommended for great surgical procedures (myocardial revascularization, valve change and combined surgery) the administration of 18g of EAAI in continuous dropping form anesthetic induction for all patients, no matter their weight, height or other biometric parameters.

The nurse manager of processes concerning heart surgery was responsible to collect and register in the institutional database demographic, epidemiologic, clinical, and laboratory parameters of each patient.

Primary endpoints included: total amount of blood products administrated intra and postoperatively, besides frequency (percentages) of EAAI administrated postoperatively. The clinical evolution was also analyzed within 30 days post cardiac surgery, and patients were divided into groups, those who underwent surgery before and after the protocol implementation. Among clinical endpoints evaluated within 30 days we included those on transfusion that were included previous studies, such as: mortality, acute renal failure (ARF), post-operative infection, septic shock, duration of hospital stay and readmission. These endpoints were also compared in patients that received blood transfusion *versus* those who did not, and also in pre-protocol and post-protocol group.

Development of this study followed ethical aspects described in Declaration of Helsinki and American Documents. Our study was approved by the Institutional Ethical and Research Committee (process # 13516613.4.0000.5533).

For statistical analyses a maximum limit of significance of 5% was defined for chance of type I error (p<0.05) in two-tailed tests. Continuous variables were presented as means and standard deviation for cases that proximity could be determined with a normal distribution. The Student's t test was used to compare proximities. The Wilcoxon and Mann-Whitney tests were applied to other continuous variables; presented by means and interquartile ranges (25th and 75th percentile). Data related with categorical variables were analyzed using the χ^2^ test or the Fisher exact test when necessary. Statistical calculations were done using the Statistical Package for Social Sciences program (SPSS, Chicago, Illinois, USA) version 20.0.

## RESULTS

In 2011, 533 patients underwent great cardiac surgery at *Hospital TotalCor*. From this total, 67% were men with mean age of 59 years old and in 5% of them ECC was not used. Myocardial revascularization surgery accounted for 61% (n=326) of procedures. Our study analyzed this group concerning effects of interventions related with the rational transfusion protocol. Valve surgeries were conducted in 15% of patients (n=81) from all cardiac surgeries whereas the remained 24% (n=126) were considered as other surgeries (combined, congenital, aorta, etc.). Characteristics of patients who underwent myocardial revascularization surgery were analyzed and, among them, 8% (n=25) did not used ECC whereas 92% (n=301) used ECC in the procedure (Table 1). The protocol for the use of blood products was implemented between April and June. Demographic characteristics of patients who underwent myocardial revascularization before and after protocol implementation are described in table 1.

**Table 1:** General characteristics of the two groups

Characteristics	Myocardial revascularization 2011 (n=326) January to December	Group 1 (n=77) January to March	Group 2 (n=174) July to December	p value
Mean age (years)	62	60	63	NS
Men (%)	75	73	76	NS
SH (%)	79	76	82	NS
DM (%)	38	36	37	NS
Previous AHF (%)	43	45	44	NS
Previous stroke (%)	3	3	4	NS
COPD (%)	2	2	1	NS
Dialytic ARF (%)	3	3	3	NS
Medium creatinine	1.18	1.12	1.22	NS
EF (%, mean)	58	56	59	NS
Previous TCA (%)	12	10	11	NS
Previous cardiac surgery (%)	0	2	0	NS
Emergency or urgent surgery (%)	60	55	54	NS
Use of ECC (%)	92	94	91	NS
IAB IPO (%)	3	3	4	NS

NS: not significant; SH: systemic hypertension; DM: diabetes mellitus; AHF: acute heart failure; COPD: Chronic Obstructive Pulmonary Disease; ARF: acute renal failure; EF: ejection fraction; TCA: transluminal coronary angioplasty; ECC: extra-corporal circulation; IAB: intraaortic balloon; IPO: immediate postoperative.

**Figure 1:**
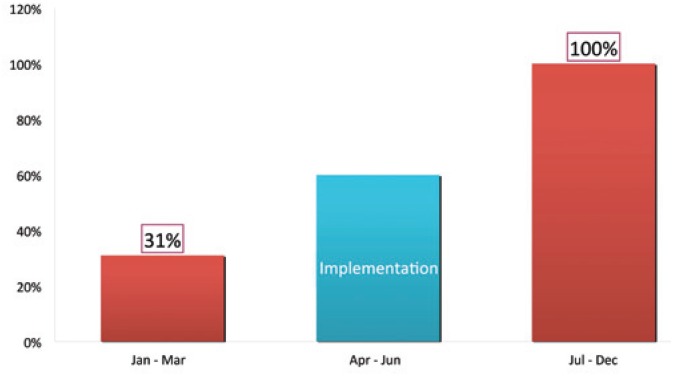
Use of ε-aminocaproic acid before and after protocol implementation

**Figure 2:**
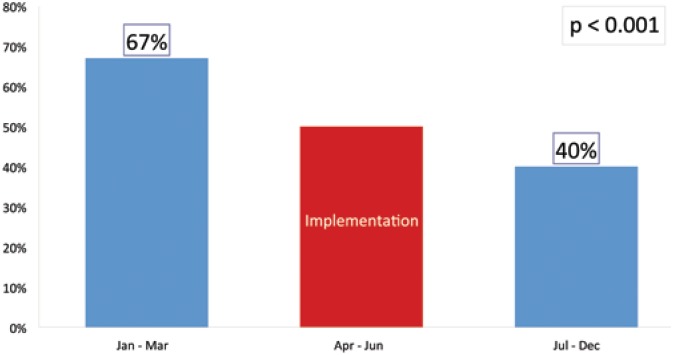
Effect of protocol implementation for rational use of blood products on total of blood transfusions

Effects of the educative interventions concerning blood transfusion protocol after its implementation showed that EAAI rose from 31% to 100% (Figure 1). During subsequent months of the same year we also observed that before protocol implementation 67% of surgeries required transfusion, however, after that transfusions were needed in 40% (Figure 2), p<0,001.

Of patients who underwent coronary artery bypass graft surgery in 2011, 151 (46%) received blood components in perioperative and of transfused patients, 93% received concentration of erythrocytes, 22.5% fresh frozen plasma and 14% concentration of platelets. After the protocol implementation a reduction was seen in used of three blood products, with a decrease of 10% in the use of fresh frozen plasma and concentrated platelets (Table 2).

**Table 2:** Use of blood products pre- and post-implementation

Blood product	Pre-implementation (%)	Post-implementation (%)	p value
Fresh plasma	20	7	<0.001
Platelets	15	4	<0.001
Concentration of erythrocytes	64	36	<0.001

Complications recorded in the study follow-up period (Table 3) shown that they differed between groups that received and did not receive blood transfusions. The ARF was observed in 11% of transfused patients *versus* 1% of non-transfused patients (p<0.0001); postoperative infection occurred in 23% of patients who received blood products *versus* 14% of those who did not (p=0.04); Septic shock was seen in 3% of transfused patients *versus* 0% of non-transfused individuals (p=0.02). The mean hospital stay in the intensive care unit and hospital stay was longer in the transfused group (3.3 and 7.3 days, respectively) compared with the non-transfused group (2.8 and 6.9 days, respectively). A total of 17% of transfused patients were readmitted, and only 10% of those who did not receive blood products were readmitted (p=0.05). Mortality rate of transfused group was 2% *versus* 0% in non-transfused group (p=0.10).

**Table 3:** Clinical endpoints according to use of blood products

	Group 1 (n=151)	Group 2 (n=175)	p value
ARF (%)	11	1	<0.0001
Infection (%)	23	13	0.04
Septic shock (%)	3	0	0.02
Duration of stay in ICU in days (SD)	3.2 (2)	2.4 (1.9)	0.003
Mean of postoperative period in days (SD)	7.3 (3.7)	6.3 (3.7)	0.02
Readmissions (%)	17	10	0.05
Mortality rate (%)	2	0	0.10

Group 1 received blood products; Group 2 did not received blood products.

ARF: acute renal failure; ICU: intensive care unit; SD: standard deviation.

Clinical endpoints that presented higher frequency in transfused patients were monitored in both groups before and after protocol implementation (Table 4). Our results identified improvement in most of parameters after the educative intervention, i.e., a reduction of ARF cases after protocol implementation (9% before *versus* 4% after), however, no statistical significance was seen (p=0.13). Therefore, no statistically significance difference was seen in groups before and after protocol implementation in endpoints evaluated (Table 4).

**Table 4:** Clinical endpoints pre and after protocol implementation

	Group 1 (n=77) January to March	Group 2 (n=174) July to December	p value
ARF (%)	9	4	NS
Infection (%)	19	20	NS
Septic shock (%)	2	2	NS
Duration of stay in ICU (mean; medium – days)	2.9; 2	2.7; 2 days	NS
Intra and postoperative hospital stay (mean; medium – days)	6.9; 6	7.0; 6	NS
Readmissions (%)	18	15	NS
Mortality rate (%)	3	3	NS

ARF: acute renal failure; ICU: intensive care unit.

## DISCUSSION

This study enabled to observe that an institutional protocol implementation of adequate practices in blood transfusions led to a considerable reduction in the use of blood products in perioperative period in individuals who underwent cardiac surgery. In secondary endpoints, we observed that transfusion group had higher incidence of clinical complications, however, the reduction in the use of blood products after the protocol, was not associated with a significant difference in evaluated clinical parameters.

Basal characteristics of population were similar and no significant variation was found among prognosis factors evaluated in groups before and after begin the protocol for rational use of blood products.

It was observed that number and frequency of perioperative complications in operated patients were higher in those who received blood products. This data is not new, especially because previous studies described that blood transfusions are related not only with more surgical complications, but also with the increase in mortality^(17–21)^, both immediately after the procedure and in long-term period^(22,23)^. The mortality assessment did not show statistical significance in our analysis, probably for the small size of sample and, mainly, for the low mortality in population who composed the study.

The rational use of blood products using educative intervention based on an institutional protocol associated with infusion of Ɛ-aminocaproic acid reduced blood transfusions in postoperative of cardiac surgery, which can affect the risk of complications. In our sample there was low percentage of ARF cases and readmissions after the protocol implementation, however, this difference did not show statistical significance. The others clinical endpoints such as hospital stay, infections and mortality in postoperative also not presented statistically significant changes. Literature reviews^(24,25)^ suggest that a more conservative strategy in transfusion is not associated with worse endpoints.

Randomized clinical trials that limit the use of blood products in critical patients^(26)^ and, particularly, in post-cardiac surgery patients^(14)^ showed more trustable results concerning the lack of difference in reduction of major clinical endpoints. Therefore, our results reinforce such evidences. Although, subgroup analyses^(26)^ showed that younger patients had low risk when included in the strategy of transfusion restriction, they also had better endpoints than those with similar characteristics, that is, who received transfusion in a more liberal manner.

Despite the potential clinical benefit in specific subgroups, the advantage of the chosen strategy in general population was the finding that the number of transfusion can be reduced without compromise the clinical endpoints in postoperative of cardiac surgery. This evidence is important to reinforce the strategy aim in the use blood products in an optimized manner, therefore, avoiding unnecessary transfusions, and reducing costs and possible clinical complications^(26–27)^.

Our study limitations are the fact that it was conducted at a single center and was composed by a small sample. Also, the lack of randomized control group turned the analysis of endpoints vulnerable to confusion factors. Adjustments made towards potential confusion factors reduced this possible bias, however, unknown prognosis factors or that were included in calculation would influence results concerning statistical adjustment. In addition, prognosis differences are more important in comparisons among patients who received transfusion with those who did not, which was not the main objective of this study. Groups compared in our study were patients who underwent myocardial revascularization before and after educative interventions. Both groups included had similar prognosis characteristics. The significant reduction of transfusion after protocol implementation was not associated with worsening in clinical endpoints. Although, this observation was made within a context of retrospective analysis we can state over observation of data on clinically similar patients – treated in same service and by the same medical team – that effect of educative interventions was consistent to reduce the use of blood products and showed safety concerning risks of complications.

## CONCLUSION

A protocol implementation to encourage the rational use of blood products was associated with the reduction of blood transfusion in perioperative of myocardial revascularization. Patients who were treated based on the protocol had good clinical evolution in the postoperative period.
